# Active and Intelligent Packaging for Enhancing Modified Atmospheres and Monitoring Quality and Shelf Life of Packed Gilthead Seabream Fillets at Isothermal and Variable Temperature Conditions

**DOI:** 10.3390/foods11152245

**Published:** 2022-07-27

**Authors:** Maria Katsouli, Ioanna Semenoglou, Mado Kotsiri, Eleni Gogou, Theofania Tsironi, Petros Taoukis

**Affiliations:** 1 Laboratory of Food Chemistry and Technology, School of Chemical Engineering, National Technical University of Athens (NTUA), 15780 Athens, Greece; mkatsouli@chemeng.ntua.gr (M.K.); isemen@chemeng.ntua.gr (I.S.); elenigogou@uth.gr (E.G.); ftsironi@aua.gr (T.T.); 2 Institute of Marine Biology, Biotechnology and Aquaculture, Hellenic Centre for Marine Research, 46.7 Athens-Sounio Ave, 19013 Attiki, Greece; mkotsiri@hcmr.gr; 3 Department of Food Science and Nutrition, University of Thessaly, End N. Temponera, 43100 Karditsa, Greece; 4 Laboratory of Food Process Engineering, Department of Food Science and Human Nutrition, Agricultural University of Athens, 11855 Athens, Greece

**Keywords:** modified atmosphere packaging, active packaging, CO_2_ emitters, Time Temperature Integrators, intelligent packaging, fish fillet, shelf-life modeling, volatiles production, freshness

## Abstract

The study investigated the effect of active modified atmosphere packaging (20% CO_2_–60% N_2_–20% O_2_) with CO_2_ emitters (MAP-PAD) and conventional MAP (MAP) on the quality and shelf-life of gilthead seabream fillets during chill storage, while the most appropriate enzymatic Time Temperature Integrators (TTI) were selected for monitoring their shelf-life at isothermal and variable temperature storage conditions (*T_eff_* = 4.8 °C). The concentration of CO_2_ and O_2_ in the headspace of the package, volatile compounds and of the microbial population were monitored during storage. The kinetic parameters for bacterial growth were estimated at 0–10 °C using the Baranyi growth model. The MAP-PAD samples presented significantly lower microbial growth rates and longer lag phases compared to the MAP samples, leading to significant shelf-life extension: 2 days of extension at 2.5 °C and 5 °C, while 50% extension at variable conditions (*T_eff_* = 4.8 °C). CO_2_ emitters in the package improved the chemical freshness (K-values) and volatile compounds (characterizing freshness). The responses of different enzymatic TTI were modeled as the function of enzyme concentration, temperature and storage time. The activation energy (*E_a_*) ranged from 97 to 148 kJ mol^−1^, allowing the selection of appropriate TTIs for the shelf-life monitoring of each fish product: LP-150U for the MAP and M-25U for the MAP-PAD samples. The validation experiment at *T_eff_* = 4.8 °C confirmed the applicability of Arrhenius-type models, as well as the use of TTIs as effective chill chain management tools during distribution and storage.

## 1. Introduction

Consumers lean towards the consumption of fresh fish products, as they contain a variety of essential elements (i.e., proteins, vitamins, nutrients and long-chain polyunsaturated fatty acids) [1]. However, raw fish and, especially, fresh fillets are highly perishable and deteriorate rapidly due to a combination of microbial breakdown mechanisms, autolytic biochemical reactions and chemical oxidation of lipids, even when they are stored in refrigerated conditions [2]. The shelf-life of seafood products ranges from 2 to 10 days, depending on the species, initial bacterial load, harvest location and season, packaging method and temperature conditions during transportation and storage [1]. The limited and variable shelf-life of chilled fish creates a major problem for quality assurance and commercial viability; thus, the application of new preservation methods for shelf-life extension is appropriate [3].

Proper packaging improves the shelf-life and maintains the high quality of fish, enhancing consumer acceptability and, hence, marketability. Alteration of the headspace gas composition can extend the shelf-life of refrigerated fish, which can be achieved by vacuum packaging or modified atmosphere packaging (MAP). MAP has been proven as an effective technology to maintain fish products’ quality using a specific combination of various gases, such as nitrogen, carbon dioxide and oxygen, as well as at various concentrations in the package headspace. This technology delays microbial growth, retarding the formation of chemical compounds, such as trimethylamine (TMA) and total volatile base nitrogen (TVBN), or sustenance of the sensorial characteristics [3,4,5]. According to the literature, the preservative effect of MAP on fish results in a shelf-life extension of several days, depending on the species and storage conditions; specifically, MAP combined with low storage temperatures has been reported as a highly effective technology [4,6,7]. 

It is well-established that the bacterial growth in fish products can be reduced proportionally to the amount of dissolved CO_2_ in the fish flesh. Various researchers have established that the CO_2_ concentration inhibits respiratory organisms’ growth, i.e., *Pseudomonas* spp. and *Shewanella* putrefaciens; the higher the concentration in the system, the higher the inhibitory effect [4,6]. This inhibitory effect depends on four complex mechanisms, such as effects on the nutrient uptake and absorption of the cell membrane functions, decreases or inhibition of the rates of enzyme reactions, intracellular pH changes that may occur by the penetration of bacterial membranes and direct physicochemical changes in the properties of proteins [6]. 

Despite the advantages of MAP, it is space-demanding, as a high-gas volume/product volume ratio (g/p) is needed in order to preserve the high quality of fish products. However, the concentration of CO_2_ within the package will change due to the partial dissolution of CO_2_ into the product and permeability through the packaging film [8,9]. The use of CO_2_ emitters (pads) inside the modified atmosphere packaging can be viewed as a technique complimentary to MAP to overcome the drawbacks. Carbon dioxide emitters utilizing the O_2_ in the package headspace produce CO_2_, thus enhancing the concentration of CO_2_/N_2_ inside the package headspace without needing an initial high CO_2_ concentration. The addition of CO_2_ emitters in the bottom of the tray can compensate for an initial low g/p ratio after sealing the package, thereby preventing the requirement of an initial high g/p and the deformation of the package during storage [3,8,9]. Moreover, these pads produce CO_2_ when they come into contact with the water leaked from the food matrix; thus, they may also simultaneously act as liquid absorbers [7]. Minimizing the liquid loss during storage is important in order to obtain an attractive product for the consumers, prevent microbial growth due to an excess of water in the container and avoid sensory quality loss because of reduced juiciness [1]. This type of active packaging is an innovative way to maintain the gas composition throughout the storage period and to extend the product’s shelf-life while preserving the quality of the packaged food [8]. 

A substantial portion of chilled fish products is exposed throughout distribution to a wide range of storage temperatures far beyond the optimal conditions [10,11]. Thus, effective control of the chilled distribution of fresh fish products is vital to their commercial viability. The application of a quality and safety assurance system for chilled fish products is required in order to continuously monitor the storage conditions from production to consumption [10,12]. Direct (e.g., freshness indicators) or indirect (e.g., Time Temperature Integrators) information reflecting the quality of the fish product during storage can be provided by using a smart packaging system. Time Temperature Integrators (TTI) are smart labels designed to monitor the food product temperature history and reflect the quality throughout the chilled chain. The operation of TTI is based on mechanical, chemical, electrochemical, enzymatic or microbiological changes, usually expressed as a visible response in the form of a mechanical deformation, color development or movement [11,13]. By using reliable models of the shelf-life of both packed fish and the TTI response, the effect of temperature can be quantitatively translated to the food quality, from production to consumption [14]. The data obtained from the TTI response at designated points of the chilled chain could lead to realistic control of the chill chain, resulting in an effective reduction of waste and efficient shelf-life management [15].

Various researchers have studied the effect of the CO_2_ emitter as an effective tool complementary to MAP in order to extent the shelf-life of Mediterranean farmed gilthead seabream (*Sparus aurata*) and sea bass (*Dicentrarchus labrax*) during refrigerator storage [8,14,16,17]. Moreover, different studies have focused on the appropriate selection of different types of TTI with fish products, but there is a lack of published data for the combination of enzymatic TTI with packed seabream (*Sparus aurata*) fillets [12,14,15,18]. However, limited studies have focused on active and intelligent packaging systems by incorporating active tools (CO_2_ emitter) and smart functionalities (TTI labels) simultaneously into one system. The validated the shelf-life models for active and smart MAP fish products provide useful information on the quality of packed gilthead seabream fillets in the actual chill chain and on independent fish batches. 

Thus, the study aims to evaluate the effect of CO_2_ emitters as MAP enhancers on the microbial stability and shelf-life of gilthead seabream fillet and also the selection of the most suitable TTI smart labels for monitoring the shelf-life of packed fish fillets.

## 2. Materials and Methods

### 2.1. Kinetic Study of Quality Deterioration of Gilthead Seabream Fillets

#### 2.1.1. Raw Material and Packaging

**Figure 1:**
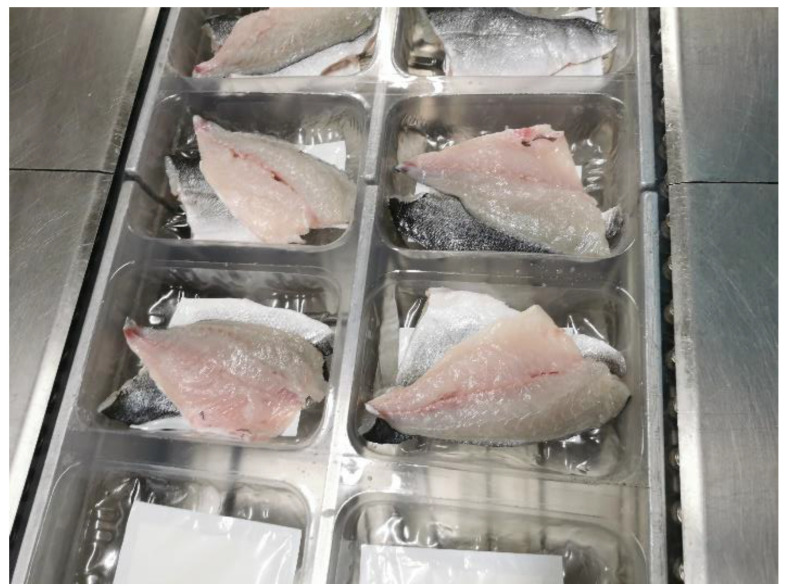
Packaging of gilthead seabream fillets in a thermoforming machine for modified atmosphere packaging with a CO_2_ emitter pad.

Fresh marine cultured gilthead seabream (*Sparus aurata*) fillets were provided by Avramar S.A., a Greek aquaculture company (capture zone: Aegean Sea, Greece). After harvesting, the fish was transported in ice (0 °C) to the industrial processing line of the company within 24 h. Fish were scaled, filleted (weight: 90–120 g per fillet) and rinsed with water. Fish fillets were packed (2 fillets per package) under modified atmosphere (gas composition: 20% CO_2_, 20% O_2_ and 60% N_2_) and transferred directly in the Laboratory of Food Chemistry and Technology (NTUA). Both the used tray and the upper plastic filmwere made with Polyethylene Terephthalate (PET) material. The samples packed under modified atmospheres were coded as MAP, while in half the number of samples, a CO_2_ emitter was placed at the bottom of the tray (coded as MAP-PAD, Figure 1). The fillets were placed with the fish skin in contact with the emitter. The CO_2_ emitters with dimensions: 80 × 130 mm, maximum absorbency: 35 mL, maximum CO_2_ production: 120 mL were provided by McAirlaid’s (Steinfurt, Germany). Changes of the gas headspace in the packaging during storage were determined using the CheckMate 9900 O_2_/CO_2_ m (PBI Dansensor, Rinsted, Denmark).

#### 2.1.2. Kinetic Study of Microbial Growth

Samples were stored in high-precision (±0.2 °C) low-temperature incubators (Sanyo MIR 153, Sanyo Electric, Ora-Gun, Gunma, Japan) at isothermal conditions (0 °C, 2.5 °C, 5 °C and 10 °C). The incubator temperature was monitored through programmable data loggers (COX TRACER^®^, Belmont, NC, USA). Two replicate samples were tested each time. The evaluation of the quality deterioration of gilthead seabream fillets was based on microbial growth and the concentration of CO_2_ and O_2_ in the package headspace. More precisely, microbial growth was based on enumeration of the total viable count (TVC) in *Pseudomonas* spp., *Enterobacteriaceae* spp. and H_2_S-producing bacteria (e.g., *Shewanella* spp.).

A microbiological analysis was performed according to Tsironi et al. [8]. Ten grams of gilthead seabream fillets were mixed with 90 mL of sterilized Ringer solution (Merck, Darmstadt, Germany) and were transferred to a sterile stomacher bag in order to be homogenized for 1 min with a Stomacher (BagMixer ^®^ interscience, Cantal, France). Different 10-fold serial dilutions were prepared in the Ringer’s solution. Two replicates of three different appropriate dilutions were prepared at each microorganism tested. For the enumeration of TVC and *Pseudomonas* spp., samples of 0.1 mL of different dilutions were spread on the count agar (PCA, Merck, Darmstadt, Germany) for TVC and on cetrimide agar (CFC, Merck, Darmstadt, Germany) for *Pseudomonas* spp., and their colonies were enumerated after incubation at 25 °C for 72 h and 48 h, respectively. In the case of *Enterobacteriaceae* spp. and H_2_S-producing bacteria, the pour plate method was used. *Enterobacteriaceae* spp. were grown on violet, red bile glucose agar (VRBG, Merck, Darmstadt, Germany) and incubated at 37 °C for 24 h, and H_2_S-producing bacteria were grown on iron agar with L-cysteine (iron agar, Merck, Darmstadt, Germany) and incubated at 25 °C for 48 h.

The Baranyi growth model was used to describe the microbial growth for each microorganism tested at all the examined conditions in order to estimate the growth rate (*k*), the lag phase (*λ*) and the maximum microbial population (N_max_) [19]. In this framework, the DMfit program (IFR, Institute of Food Research, Reading, UK) was used (available at http://www.combase.cc/index.php/en/, accessed on 15 March 2022) for curve fitting. The temperature dependence of the growth rate constants was modeled via the Arrhenius Equation (1):

(1) $$lnk=lnk_{ref}-\left(\frac{E_{a}}{R}\right)\left(\frac{1}{T}-\frac{1}{T_{ref}}\right)$$

where *k_ref_*: growth rate constant of the microorganism at a reference temperature, *T_ref_*_:_ equal to 4 °C, *T*: temperature in K, *E_a_*: activation energy of the studied action, which indicates the temperature dependance and *R*: universal gas constant (R = 8.314 J/(mol∙K)). 

#### 2.1.3. Freshness Assessment and Volatiles Production

Freshness assessment of gilthead seabream fillets was based on an ATP breakdown products analysis (K-values index) and on the volatile compound (VOC) contents. Extraction of ATP breakdown products took place from the dorsal muscle. The extraction of ATP breakdown products and the HPLC K-values analysis has been previously described [20]. The breakdown of ATP into inosin (Ino) and hypoxanthine (Hx) was used to define a K-value, based on the ratio between the concentration of Hx, Ino and the intermediates compounds (ATP metabolites). For each compound, their ATP-related compounds were calculated based on the peak areas using the external standard. 

A method for VOC determination by headspace SPME-GC/MS analysis was developed according to Parlapani et al. [16]. Specifically, volatiles were extracted by homogenizing 5 g of minced fish muscle with 4 mL of saturated saline solution in a 15-mL glass vial fitted with a PTFE/silicone septum. The solution was incubated under stirring at 40 °C for 15 min to equilibrate. The SPME fiber (50/30UM DVB/CARBOXEN-PD) was exposed to the headspace for an additional 40 min under the same conditions. 4-methyl-1-pentanol was used as the internal standard. The headspace was then analyzed using gas chromatography-mass spectrometry (Agilent Technologies, Santa Clara, CA, USA), and the separation was achieved on an Agilent DB-WAX GC Column (30 m 0.25 mm, coated with a 0.25-μm film thickness). The injection port was equipped with a liner (78.5 mm × 6.5 mm × 0.75 mm) suitable for SPME analysis. The oven temperature was initially maintained at 40 °C for 2 min, then was raised to 100 °C at a rate of 5 °C/min, 110 °C at a rate of 1 °C/min, 150 °C at a rate of 10 °C/min and, finally, was raised to 240 °C at a rate of 10 °C/min and held for 5 min. The injector was operated in the split mode, and the temperatures of the MS source and quadrupole were set at 230 and 150 °C, respectively. Identification of the compounds was based on comparing MS data with those of the reference compounds and by MS data obtained from the NIST library (NIST/EPA/NIH Mass Spectral Library with Search Program, software version 2.0f) and by semi-quantitative analysis using the method of internal standard.

#### 2.1.4. Shelf-Life Estimation (*t_SL_*)

The shelf-life estimation was calculated using Equation (2):

(2) tSL=logN1−logNokrefexp−EaR(1T−1Tref)+λrefexp−EaR1T−1Tref

where *t_SL_*: estimated shelf-life in days, $logN_{1}$: limit of total viable counts load (7 log CFU g^−1^), according to the literature [21,22,23], $logN_{0}$: initial value of the total viable counts, *k_ref_*: growth rate constant of the microorganism at a reference temperature, *T_ref_* = 4 °C, *T*: temperature in K, *E_a_*: activation energy of the studied action, which indicates the temperature-dependance, *R*: universal gas constant (R = 8.314 J/(mol·K)) and *λ*: lag phase. 

### 2.2. Kinetic Study of Time Temperature Integrators Response

#### 2.2.1. Time Temperature Integrators

**Figure 2:**
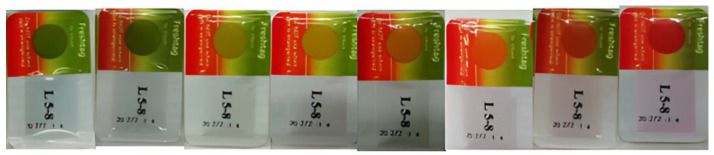
Response scale of enzymatic FreshTag TTI.

The enzymatic FreshTag TTI were provided by Vitsab A.B. (Malmo, Sweden) and based on an enzymatic hydrolysis of a lipid substrate, which is time–temperature-dependent. After TTI activation, the enzyme and substrate were mixed, and the hydrolysis resulted in a controlled pH decrease, which corresponds to a color change from green to yellow, orange and, finally, to red (Figure 2). Two different types of enzymatic indicators were examined, M-type, which consist of methyl myristate as the substrate and LP-type with a mixture of trilaurin and tripalmitin, while both types contained lipase from *Rhizopus oryzae*. The concentration of this enzyme ranged from 5 to 100 units for M-type and from 100 to 500 units for LP-type TTI in order to achieve a variety of response times.

All the examined TTI were stored isothermally at 0, 2.5, 5, 10 and 15 °C in high-precision (±0.2 °C), low-temperature incubators (Sanyo MIR 153, Sanyo Electric, Ora-Gun, Gunma, Japan) and the incubator temperatures were monitored through programmable COX TRACER^®^ data loggers (Belmont, NC, USA). The TTI response was based on color measurements using the X-Rite i1 Pro (X-rite, Grand Rapids, MI, USA) spectrophotometer and the CIELab color scale at D_50_ illumination. For each TTI type, 5 TTI labels were measured at appropriate time internals for all isothermal storage conditions. The TTI response was described by the normalized value (*a* + *b*), as shown in Equation (3) [24]:

(3) $$norm\left(a+b\right)=\frac{\left(a+b\right)-{\left(a+b\right)}_{min}}{{\left(a+b\right)}_{max}-{\left(a+b\right)}_{min}}$$

where *a* and *b*: CIELab color scale parameters, $\left(a+b\right)$: the measuring value of the sum at each time, ${\left(a+b\right)}_{min}$: the minimum value of the sum of these parameters and ${\left(a+b\right)}_{max}$: the maximum value. More precisely, *a* represents the green–red colors, with negative values (up to −60) toward green and positive values (up to +60) toward red. In the case of b, values up to −60 represent blue, and positive values up to 60 represent yellow. 

#### 2.2.2. Kinetic Modeling of TTI Response

The TTI response to the storage time could be fitted in a sigmoidal curve described by the logistic equation of Equation (4):

(4) $$norm\left(a+b\right)=\frac{1}{1+exp\left(\frac{k_{1}-t}{k_{2}}\right)}$$

where *k*_1_ and *k*_2_: response rate constants depending on the enzyme concentration and temperature, respectively. The value 1/*k*_2_ is the phase’s slope in which the TTI response changes exponentially with time [24,25]. These parameters were calculated for each TTI label and storage temperature using a nonlinear regression analysis (Sigmaplot 12.0, Systat Software Inc., Palo Alto, CA, USA).

The response ranged from 0 for green to 1 for red. Based on the manufacturer (VITSAB A.B.), the visual end point of the TTI corresponds to an orange–red color, which was found to be equal to 0.8 based on the instrumental color measurements. In addition, this end point represents the end of the exponential phase of Equation (3). 

The response rate constants of Equation (3) depend on the temperature, which is expressed through the activation energy (E_a_) of the Arrhenius equation (Equation (1)). A total mathematical model (Equation (5)) has been developed for each type of TTI (M-type and LP-type), which describes the dependence of the TTI response from the enzyme concentration (*C*), temperature (*T*) and time (*t*).

(5) $$norm\left(a+b\right)=\frac{1}{1+exp\left(\frac{k_{1ref\left(C=1U\right)}\cdot C^{-a}\cdot exp\left[\frac{-E_{a}}{R}\left(\frac{1}{T}-\frac{1}{T_{ref}}\right)\right]-t}{k_{2ref\left(C=1U\right)}\cdot C^{-b}\cdot exp\left[\frac{-E_{a}}{R}\left(\frac{1}{T}-\frac{1}{T_{ref}}\right)\right]}\right)}$$

where *k_1ref(C=1U)_* and *k_2ref(C=1U_*_)_: the response rate constants for a TTI with 1 unit enzyme concentration at the reference temperature *T_ref_* (equal to 4 °C) and *a* and *b* constants, *E_a_*: activation energy, *R*: universal gas constant (*R* = 8.314 J mol^−1^∙K^−1^), *C*: the enzyme concentration, *T*: storage temperature (in K) and *t*: storage time (in d).

### 2.3. Selection of Appropriate TTI and Validation at Variable Temperature Conditions

In order to select an appropriate enzymatic TTI for each one of the tested fish products, the kinetic data of the fish quality deterioration and TTI response were combined to obtain an adequate match. More precisely, the shelf-life of the product and the response time of the TTI should be similar in a temperature range in which fish can be exposed during distribution in the cold chain (0–10 °C). In addition, *E_a_* value of the TTI response rate should be within ±20 kJ mol^−1^ of the *E_a_* of the quality deterioration rate of the target food product [13].

**Figure 3:**
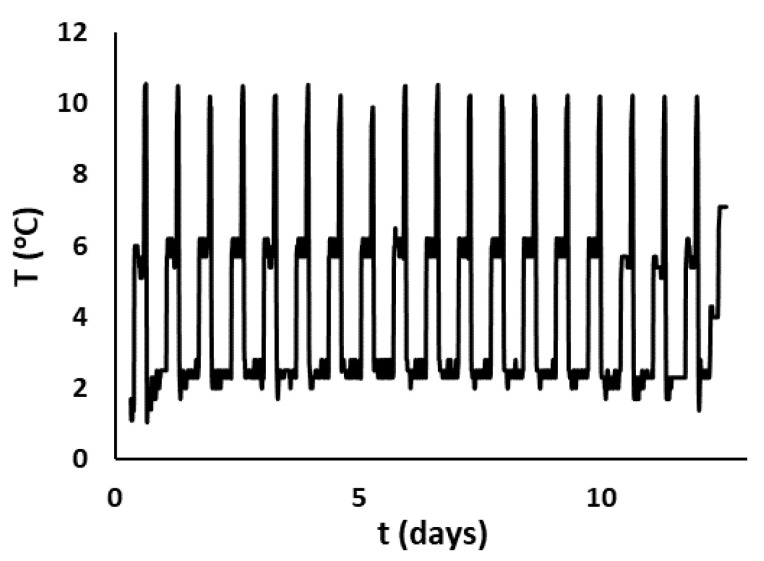
Temperature profile at variable conditions applied during the validation test.

In order to confirm the applicability of the selected TTIs and the developed Arrhenius-type models at isothermal conditions, an independent validation test was carried out at the dynamic temperature conditions, which more adequately describe the temperature variations of the actual cold chain. The MAP and MAP-PAD samples were packed as described in Section 2.1.1, and then, the selected TTIs were attached at each package. All samples were stored in programmable incubators, and the temperature was recorded through programmable data loggers (COX TRACER^®^, Belmont, NC, USA). A time–temperature scenario within the range 2–10 °C and effective temperature (*T_eff_*) equal to 4.8 °C was used for the validation experiment (Figure 3). The term *T_eff_* was introduced to describe the equivalent constant temperature that resulted the same as the variable temperature distribution at the same time period.

A microbiological analysis of the fish and color measurements of the TTI was performed according to the previously described protocols. The experimental values of the growth rates, the estimated shelf-life of the gilthead seabream fillets and the response times of the TTI were determined and compared with the respective values predicted by the developed models. 

### 2.4. Statistical Analysis

Analysis of variance (ANOVA) was performed to determine the statistically significant differences of the variables between the MAP and MAP-PAD gilthead seabream fillets. Duncan’s multiple range test at a significant level of *p* < 0.05 was applied throughout the study. The analysis was carried out using STATISTICA 7.0 (StatSoft Inc., Tulsa, OK, USA). 

## 3. Results and Discussion

### 3.1. CO_2_ and O_2_ Concentration Changes in the the Package Headspace under Isothermal Storage Conditions

**Figure 4:**
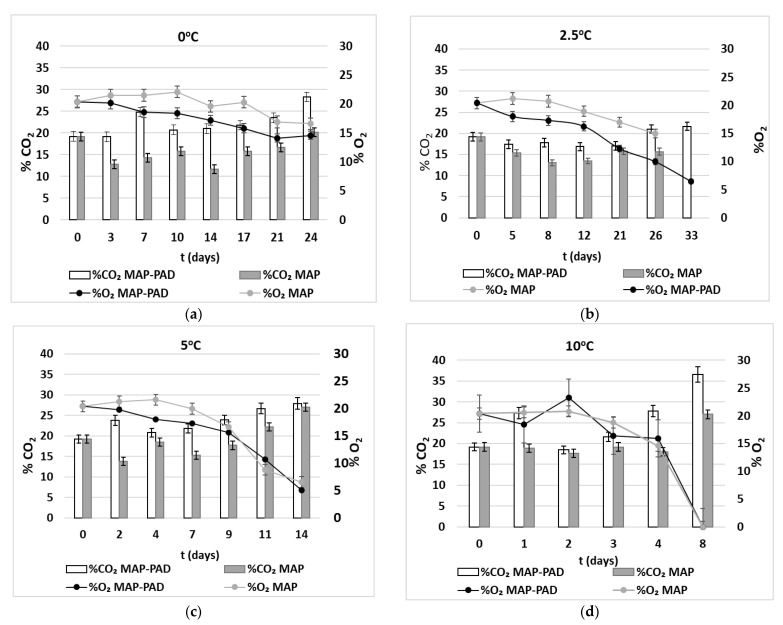
CO_2_ (bars) and O_2_ (line) concentrations in the package headspace of gilthead seabream fillets during isothermal storage at (**a**) 0 °C, (**b**) 2.5°C, (**c**) 5 °C and (**d**) 10 °C (MAP-PAD: samples in modified atmosphere packaging with C0_2_ emitters and MAP: samples in a modified atmosphere).

In the current study, during the storage period, statistically significant differences were observed regarding the concentration of CO_2_ between the MAP-PAD and MAP samples (*p* < 0.05). Specifically, the CO_2_ concentration in the headspace of the MAP samples decreased 20.0% ± 1.5%, reaching the minimum CO_2_ levels 11.6% ± 0.2%, 13.0 ± 0.5%, 13.8 ± 0.20% and 17.7 ± 0.3% on days 14, 8, 2 and 2 at 0, 2.5, 5 and 10 °C, respectively (Figure 4). Afterwards, the CO_2_ concentration increased up to the initial levels due to the metabolic activity of the bacteria involved in the spoilage of fish products. However, the CO_2_ concentration in the package headspace of the MAP-PAD samples remained relatively stable (20.71 ± 4.48%) after 17, 21, 7 and 3 days of storage at temperatures of 0, 2.5, 5 and 10 °C, respectively, while, at the end of the storage, the CO_2_ increased, reaching the maximum values (30–36% ± 2.5–1%) during all isothermal storage conditions (*p* < 0.05). Differences between the CO_2_ concentrations by temperature were also observed; samples kept at 0 °C showed greater diminution in the CO_2_ concentration than those at 5 or 10 °C (*p* < 0.05). It can be concluded that lower temperatures enhanced the CO_2_ dissolution in the fish flesh. These results agreed with the bibliography findings, as both Tsironi et al. [1] and Provincial et al. [8] observed that fish flesh absorbed higher CO_2_ at 0 °C, compared to those packed at 4 °C, 5 °C and 10 °C. The addition of a CO_2_ emitter on the tray prevented under-pressure and even increased the CO_2_ levels above the initial concentration throughout the storage period, as the pad started to produce CO_2_ as the ingredients (NaHCO_3_ and citric acid) of the emitter came into contact with the water leaked from the fish flesh (liquid loss). The CO_2_ emitters used in the present study also acted as liquid absorbent pads, making the product more attractive to consumers in terms of appearance, as, in the bottom of the tray, the liquid loss will not be noticeable to the consumers. The liquid loss of the fish products accumulating at the bottom of a tray further promotes microbial growth [9].

The O_2_ concentration was decreasing continually inside the package during storage for both the MAP and MAP-PAD samples at all isothermal temperatures, with higher temperature storage conditions amplifying the O_2_ diminution (*p* < 0.05). However, the descending trend of O_2_ for the MAP-PAD samples was higher compared to the MAP samples (*p* < 0.05). Reaching the end of the storage period, the O_2_ level was zero, which can be correlated with the augmented bacterial population of the TVC, as the bacterial respiration consumed O_2_, forming CO_2_. 

### 3.2. Bacterial Growth during Isothermal Storage Conditions—Shelf-Life Estimation

In Table 1, the initial microbial counts (log CFU g^−1^), a representative value during storage and the microbial counts at the end of storage period are presented for the TVC, *Pseudomonas* spp., *Enterobacteriaceae* spp. and H_2_S-producing bacteria, i.e., *Shewanella* spp. at 0–10 °C. In this study, the initial counts of the TVC, *Pseudomonas* spp., *Enterobacteriaceae* spp. and H_2_S-producing bacteria were higher than the reported values for gilthead seabream fillets in modified atmosphere packaging [26,27]. The initial TVC for the fresh fish fillets was approximately 5 log CFU g^−1^, and the initial H_2_S-producing bacteria counts ranged within 3.5–4.2 log CFU g^−1^. The initial *Pseudomonas* spp. counts for gilthead seabream fillets have been reported to range from 3.1 to 3.9 log CFU g^−1^ [27,28,29,30], which is lower than the initial *Pseudomonas* spp. counts in the present study (i.e., 4.5–5.1 log CFU g^−1^). The initial population of *Enterobacteriaceae* spp. ranged from 2.9 to 4.0 log CFU g^−1^. 

The TVC, *Pseudomonas* spp. and H_2_S-producing bacteria had a similar growth pattern for both the MAP-PAD and MAP samples. The MAP-PAD fish fillets reached the value of 7 log CFU g^−1^ for the TVC on days 17, 13, 8 and 3 at 0, 2.5, 5 and 10 °C, respectively, whereas, for the MAP gilthead seabream fillets, the same log CFU g^−1^ value was reached earlier on days 16, 8, 6 and 3 at 0, 2.5, 5 and 10 °C, respectively. *Pseudomonas* spp. and H_2_S-producing bacteria were co-dominants, as their counts were similar to the TVC loads at the end of the shelf-life (TVC reaching 7 log CFU g^−1^). Indeed, at all isothermal storage conditions, the H_2_S-producing bacterial population, when the TVC reached the value of 7 log CFU g^−1^, was as high as the *Pseudomonas* spp. (*p* < 0.05), while the *Enterobacteriaceae* spp. reached the value 6 log CFU g^−1^ at the end of the storage period.

Specifically, for *Pseudomonas* spp. counts, the MAP-PAD seabream fillets reached the value of 6 log CFU g^−1^ on days 24, 14, 8 and 4 at 0, 2.5, 5 and 10 °C, respectively, whereas, for the MAP seabream fillets, the microbial population reached the same log CFU g^−1^ value earlier on days 17, 12, 7 and 3 at 0, 2.5, 5 and 10 °C, respectively. H_2_S-producing bacteria reached populations of 7 log CFU g^−1^ on days 16, 8 and 3 at 0, 5 and 10 °C, respectively, for the seabream fillets with active packaging, whereas, for the MAP fish fillets, the same log CFU g^−1^ value was reached earlier on days 13, 4 and 2 at 0, 5 and 10 °C, respectively. The slower growth rates of *Pseudomonas* spp. and H_2_S-producing bacteria with the use of active packaging are important, since these microorganisms produce metabolic products affecting the sensory characteristics and, especially, the odor and the appearance of fish fillets. The *Enterobacteriaceae* spp. counts of MAP-PAD seabream fillets reached the value of 6 log CFU g^−1^ approximately after 26 and 11 days at 2.5 °C and 5 °C, respectively, and the MAP samples after 13 and 7 days of storage at 2.5 °C and 5°C, respectively. 

The microbial growth rate (k), lag phase (λ) and the maximum bacterial population counts (N_max_) of the TVC, *Pseudomonas* spp., *Enterobacteriaceae* spp. and H_2_S-producing bacteria, i.e., *Shewanella* spp., for both the MAP and MAP-PAD samples stored at 0–10 °C have been estimated by fitting the microbial growth data to the Baranyi growth model (Table 2). The inhibitory effect of the CO_2_ emitter was observed for both the TVC and *Pseudomonas* spp. growth due to the increase of the lag phase in all the MAP-PAD samples; for the TVC, λ was estimated at 8.4 d for the MAP-PAD samples compared to 5.3 d for the MAP samples at 0 °C (Table 2) and 8.7 d for the MAP-PAD samples compared to 6.4 d for the MAP at the same temperature for *Pseudomonas* spp. growth. However, no significant difference was observed regarding the growth rates of the TVC, *Pseudomonas* spp., *Enterobacteriaceae* spp. and H_2_S-producing bacteria for the MAP-PAD and MAP samples at all isothermal storage conditions (*p* > 0.05), while, at 5 °C, a significant decrease of the H_2_S-producing bacterial population growth rate of the MAP-PAD samples was observed (MAP 0.695 d^−1^ to 0.339 d^−1^ MAP-PAD at 5 °C), accompanied by the increase of the lag phase (from 0 d for MAP to 5 d for MAP-PAD at 5 °C). Similarly, to the other examined microorganisms, the growth rate of the *Enterobacteriaceae* spp. increased as the temperature increased from 0 °C to 10 °C for the MAP and MAP-PAD samples (*p* < 0.05). 

Thus, the use of CO_2_ emitters is proposed as a technique complementary to MAP, which can inhibit the microbial growth and extend the shelf-life of gilthead seabream fillets. According to the literature, as the CO_2_ increases and the O_2_ decreases, the aerobic bacteria and Gram-negative bacteria growth are delayed [3,31], with even moderate-to-low CO_2_ concentrations (10–20%) resulting in inhibition of the growth of aerobic bacteria [32]. Similar CO_2_ emitters in the modified atmosphere packaging of fish (cod, salmon and sea bass) have also been reported to effectively enhance the MAR and reduce the transport volume of MAP packaging [3,9,11]. 

The effect of temperature on the microbial growth rates (k) and lag phase (λ) for the TVC, *Pseudomonas* spp., *Enterobacteriaceae* spp. and H_2_S-producing bacteria can be described by E_a_, which was estimated by the fitting of the experimental data in the Arrhenius Equation (Equation (1)). The *E_a_* for the MAP-PAD samples for the TVC, *Pseudomonas* spp., *Enterobacteriaceae* spp. and H_2_S-producing bacteria growth rate were calculated as 113, 93, 81 and 116 kJ mol^−1^, respectively and are presented in Table 3; however, between MAP-PAD and MAP, statistically significant differences samples were not observed (*p* > 0.05), based also on the ±20 kJ/mol *E_a_* range reported in the literature [33].

According to the literature, the spoilage rejection for packaged fish in modified atmospheres with a CO_2_ emitter has been identified at log 6.5–7.0 CFU g^−1^ in terms of the TVC [8,21,22]. For gilthead seabream fillets in MAP and MAP-PAD packaging, the shelf-life in days was estimated by using the developed mathematical model (Equation (2)), based on the acceptability limit of 10^7^ CFU g^−1^ regarding the TVC [8]. 

The beneficial effect of the active packaging is demonstrated in Table 4, as the gas concentration of the MAP-PAD samples resulted in a significant shelf-life extension of gilthead seabream fillets under all isothermal conditions (*p* < 0.05); specifically, the estimated shelf-life of gilthead seabream fillets was extended by 2 days at 2.5 °C, even though the initial total aerobic viable count was high (5 log CFU g^−1^). Various studies have reported the beneficial use of CO_2_ emitters in modified atmosphere packaging in different fish products, resulting in shelf-life extensions: 2–7-day extensions for cod fillets and 4–7-day extensions for gutted sea bass [7,8,34,35]. The present study highlights the use of CO_2_ emitters serving as active packaging materials in combination with modified atmosphere packaging with low or moderate CO_2_ concentrations to extend the shelf-life of gilthead seabream fillets in the chill chain. Previous studies investigated the combination of CO_2_ emitters with MAP packaging with high gas/product ratios or a moderate-to-high initial CO_2_ concentration [3,6,7,8,9,35,36]. 

### 3.3. Freshness Assessment and Volatiles Production during Isothermal Storage Conditions

**Figure 5:**
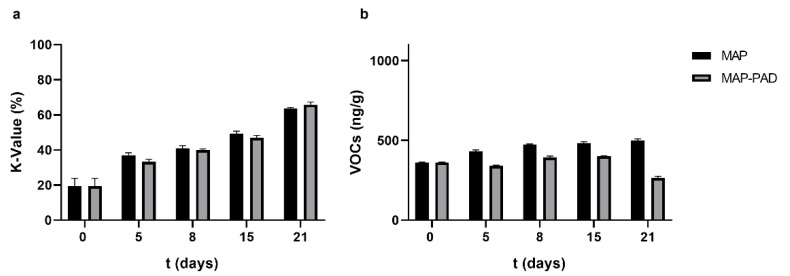
(**a**) K-value percentage alterations, and (**b**) quantitative proportion of the VOCs identified in active packaging (MAP-PAD) and modified atmosphere packaging (MAP) seabream fillets during the isothermal storage at 2.5 °C.

Using the ATP breakdown products analysis (K-values freshness index) and VOC contents, a preservation period assessment was performed for the gilthead seabream fillets stored under isothermal storage conditions in active packaging (MAP-PAD) and modified atmosphere packaging (MAP). Given that the beneficial effect of CO_2_ emitters was more prominent under storage at 2.5 °C, as the growth rate of the bacterial population significantly decreased and the lag phase significantly increased, resulting in a 2-day shelf-life extension for seabream fillets (Table 4), this temperature was chosen to be tested in the freshness quality assessment. As expected, the K-values increased over the storage period (Figure 5a); in particular, the K-values increased (initial 19% on day 0), reaching 65% on the 21st day of storage in all packaging conditions, while no statistical differences were observed with the PAD addition (*p* > 0.05) [37]. The quantitative proportion of the VOCs increased in the MAP seabream fillets during storage at 2.5 °C. The concentration of VOCs in the MAP-PAD samples was lower, especially on day 21 (Figure 5b), albeit no statistically significant differences were observed between the different packaging conditions (*p* > 0.05).

### 3.4. Kinetic Study of Time Temperature Integrators (TTI) Response at Isothermal Conditions

**Figure 6:**
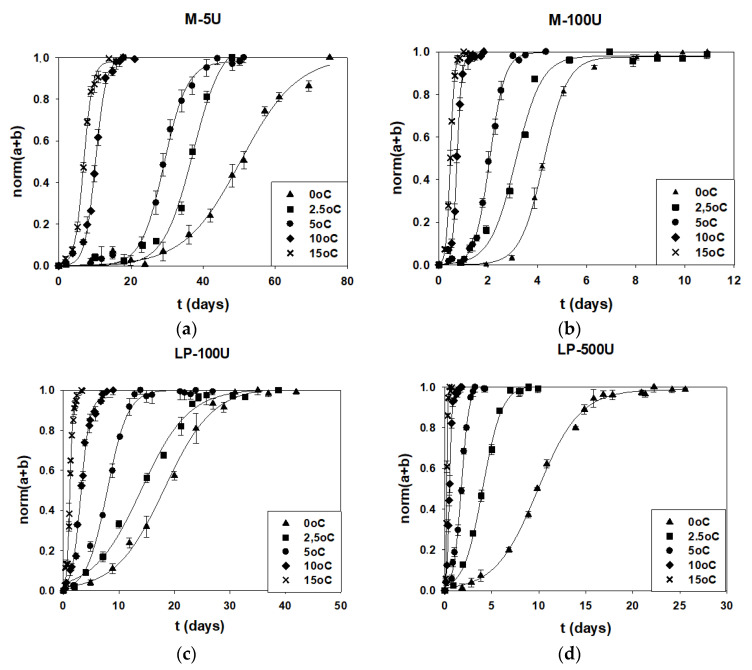
Color changes of the enzymatic TTI: (**a**) M-5U, (**b**) M-100U, (**c**) LP-100U and (**d**) LP-500U with the storage time at 0 (▲), 2.5 (■), 5 (•), 10 (◆) and 15 °C (✕).

In Figure 6a–d, the evolution of the response of the selected M-type and LP-type TTI as a function of the storage time are presented, and especially, the TTI with the lowest and the highest enzyme concentrations are depicted in the range of 0–15 °C. The response curves are similar to the curves of microbial growth, showing an exponential increase with time so they could be useful for monitoring the fish products’ quality in real time.

The rate constants of Equation (3) were calculated and are presented in Table 5 and Table 6 for the different enzymatic TTI smart labels (M- and LP-types). According to the results for each enzymatic type of the TTI, as the enzyme concentration and the storage temperature increased, the TTI responses were accelerated. The fitting of the experimental data to the models was adequate, since, at each case, the coefficient of determination (R^2^) was higher than 0.97.

A total mathematical model was calculated (Equation (4)) for each enzymatic TTI-type, determining the response *norm*(*a* + *b*) as a function of the temperature, time and enzyme concentration. The parameters of the models, which are presented in Table 7, are prerequisites for the selection of a suitable TTI for each packed sample (MAP-PAD and MAP seabream fillets). Based on the results of Table 7, LP-type TTI showed more intense temperature dependance compared to M-type, and the response rate constants *k_1ref(c=1U)_* and *k_1ref(c=1U)_* were also higher. It has been reported that temperature dependance is mainly affected by the lipid substrate, while the enzyme concentration determines the response times [38].

**Figure 7:**
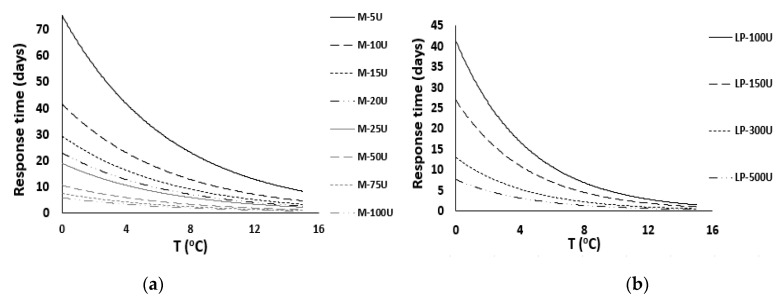
Response times of all the (**a**) M-type and (**b**) LP-type enzymatic TTIs as a function of the temperature.

Based on the total mathematical models, the shelf-life curves for all the examined TTIs were calculated and presented in Figure 7a,b.

#### Selection of Appropriate TTI for the MAP and MAP-PAD Samples

Comparing the activation energies (*E_a_*) of the examined TTI with the respective of MAP-PAD and MAP samples, they were within the range of ±20 kJ∙mol^−1^, showing that TTI can be useful for monitoring the shelf-life of seabream fillets in active and MAP packages. Apart from similar *E_a_* values, the shelf-life curves of the food and the TTI should be adequately matched. 

**Figure 8:**
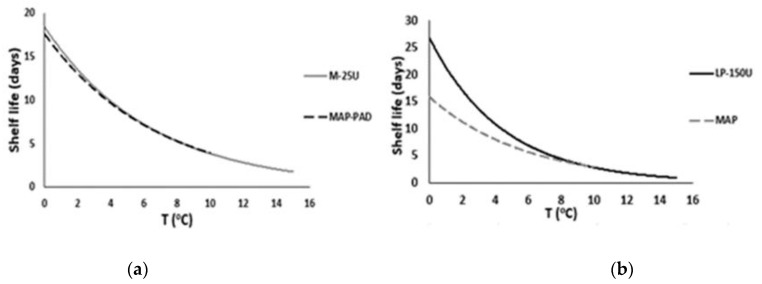
Selection of the appropriate enzymatic TTI for monitoring the shelf-life of (**a**) MAP-PAD and (**b**) MAP seabream fillets.

Applying the aforementioned criteria for the examined TTI and fish products, M-25U and LP-150U were found to be the most suitable smart labels (TTI) for monitoring the remaining shelf-life of MAP-PAD and MAP seabream fillets, respectively, as shown in Figure 8a,b. In the case of the MAP-PAD samples, an appropriate match between the fish fillet shelf-life curve and the response of the M-25U TTI was achieved. For the MAP fish fillets, LP-150U may be used conservatively for the consumers, since, at abuse temperatures (>6 °C), the TTI will expire slightly before the end of the shelf-life, while, in the recommended range, the expiration date on the food package will determine the end of the shelf-life. 

### 3.5. Bacterial Growth during Non-Isothermal Storage Conditions

The microbial growth rate (*k*), lag phase (*λ*) and the maximum bacterial population counts (N_max_) of the TVC, *Pseudomonas* spp., *Enterobacteriaceae* spp. and H_2_S-producing bacteria, i.e., *Shewanella* spp., for both MAP and MAP-PAD under non-isothermal conditions were estimated by fitting the experimental data to the Baranyi growth model (Table 8). 

*Pseudomonas* spp. and H_2_S-producing bacteria co-dominated the spoilage in both MAP and MAP-PAD gilthead seabream fillets, as their counts were similar to the total populations at end of the shelf-life (the TVC reaching 7 log CFU g^−1^). Indeed, in non-isothermal storage conditions, both bacterial populations were approximately 6 to 7 log CFU g^−1^, while *Enterobacteriaceae* spp. reached the value of 5 log CFU g^−1^. The MAP-PAD and MAP gilthead seabream fillets reached the value of 7 log CFU g^−1^ for the TVC on days 12 and 8, respectively. *Pseudomonas* spp. counts of MAP and MAP-PAD seabream fillets reached the value of 6 log CFU g^−1^ on days 11 and 15, respectively, whereas the H_2_S-producing bacteria reached the value of 7 log CFU g^−1^ on days 13 and 7 for the seabream fillets of active packaging and MAP, respectively. *Enterobacteriaceae* spp. counts of MAP-PAD and MAP seabream fillets reached the value of 6 log CFU g^−1^ after approximately 9 and 7 days, respectively. The significant inhibitory effect of the CO_2_ emitter was observed for both the TVC and H_2_S-producing bacteria as the addition of the pad significantly decreased the growth rate (*p* < 0.05), while no significant differences were observed regarding the lag phase of the MAP-PAD and MAP samples (Table 8). 

Similar to the isothermal conditions, the estimated shelf-life of seabream fillets in MAP-PAD and MAP packaging was calculated using the previously described mathematical model (Equation (2)). A significant extension of the shelf-life was observed under non-isothermal storage conditions, as the gilthead seabream fillets in the active packaging presented a 50% extension of the shelf-life (4 days of extension) compared to the MAP samples (estimated shelf-life of 8 days). This result can be attributed to the higher CO_2_ concentration in the headspace of the active package (35%) compared to the MAP samples (<25%). 

### 3.6. Freshness Assessment and Volatiles Production during Non-Isothermal Storage Conditions 

**Figure 9:**
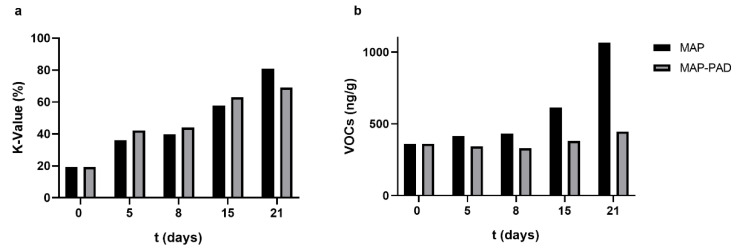
(**a**) K-value percentage alterations, and (**b**) the quantitative proportion of the VOCs identified in active packaging (MAP-PAD) and modified atmosphere packaging (MAP) seabream fillets during non-isothermal storage conditions.

As mentioned previously (see Section 3.3 above), the K-values increased over the storage period [36,39]; in particular, the K-values increased from the initial 19% on day 0 to the final values of 81% and 69% on the 21st day of storage for the MAP and MAP-PAD samples, respectively. While no statistical differences were observed between the different packages (*p* > 0.05), the PAD addition showed a slight decrease after the end of the shelf-life (Figure 9a). The quantitative proportion of the VOCs gradually increased in MAP seabream fillets during storage from 361.4 on day 0 to 1064.6 ng/g of fish muscle on day 21. Nevertheless, the addition of PAD results in a stable preservation ability during all the periods of storage; in particular, the VOCs ranged from 361.4 on day 0 to 447.1 ng/g of fish muscle on day 21 (Figure 9b). No statistically significant differences were observed between the different packaging conditions (*p* > 0.05). Volatile compound development during the non-isothermal storage conditions indicates better preservation for the MAP-PAD group. 

Taking everything into consideration, the addition of PAD improved the chemical (K-values) freshness and the total VOCs characterizing the freshness/spoilage.

### 3.7. Prediction of the for Total Viable Count (TVC), Pseudomonas spp., Enterobacteriaceae spp. and H_2_S-Producing Bacteria Growth on MAP-PAD and MAP Seabream Fillets

The developed Arrhenius-type models were validated under non-isothermal conditions. The periodically changing temperature profile, which was used for the independent validation experiment, simulated the temperature conditions of the real supply chain of the fish from production to consumption (Figure 3). The experimental data for the exponential growth rates of the TVC in gilthead seabream fillets stored in active (MAP-PAD) and MAP at non-isothermal conditions (*T_eff_* = 4.8 °C) were compared with the predicted growth rates by the developed model. Additionally, the relative errors (RE) were also calculated as RE% = ($\frac{k_{\mathrm{experimental}}-k_{predicted}}{k_{\mathrm{experimental}}})\times 100,\;$ according to the literature [14]

The predicted TVC growth rates were 0.398 ± 0.024 d^−1^ for the MAP-PAD samples and 0.524 ± 0.046 d^−1^ for the MAP samples. According to the literature, a limit of 20% was set as the criterion of applicability [14]. Given that, the model provided satisfactory predictions with 14% and 5% errors for the MAP-PAD and MAP samples, respectively, demonstrating that the developed model can adequately describe the growth of the TVC in MAP and MAP-PAD seabream fillets with the gas concentration 20% CO_2_, 60% N_2_ and 20% O_2_ under non-isothermal refrigerated storage. The developed model can also satisfactorily describe all the examined bacterial populations, as the error of prediction did not exceed the 20% limit [14], as presented in Table 9. Several predictive models for microbial growth in non-isothermal conditions have been reported in the literature [40,41,42], while a number of fish spoilage predictive models have been validated at variable temperature conditions, such as, for example, for the Mediterranean fish boque (*Boops boops*) [43] and gilthead seabream [44].

### 3.8. Validation of the Selected TTI at Non-Isothermal Storage Conditions

**Figure 10:**
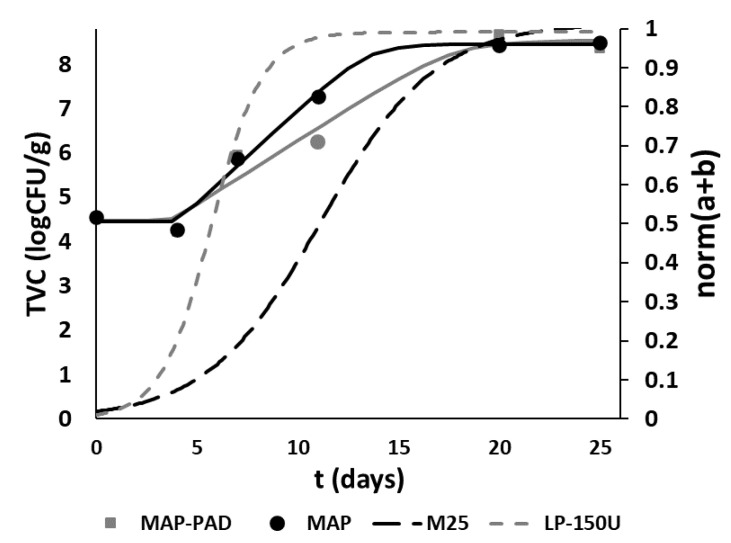
Growth curve of the total viable counts (TVC in the MAP and MAP-PAD seabream fillets (left vertical axis), and responses of the M-25U and LP-150U enzymatic TTIs (right vertical axis) under non-isothermal storage conditions (*T_eff_* = 4.8 °C).

Under non-isothermal conditions, the selection of the most suitable TTI and the applicability of the models were validated. In Figure 10, both the responses of M-25U and LP-150U and the TVC growth with the storage time are depicted. Both TTI provided satisfactory applicability; specifically, the difference between the response time of the TTI (end point at *norm*(*a* + *b*) = 0.8) and the shelf-life of the fish fillets (TVC load = 7 log CFU g^−^^1^) was 8% for the MAP-PAD seabream fillets and M-25U and 12.5% for the MAP and LP-150U. 

The experimental response rates of M-25U TTI were 11.3 ± 0.13 d^−1^ for *k*_1_ and 1.78 ± 0.08 d^−1^ for *k*_2_ and equal to the 5.71 ± 0.06 d^−1^ for *k*_1_ and 0.93 ± 0.05 d^−1^ for *k*_2_ rates of LP-150U TTI. Comparing the experimental with the predicted values, the models provided satisfactory predictions, with 14.5% and 6.9% errors for M-25U and LP-150U, respectively, in the case of k_1_ rates and 5.5% and 17.9% errors for M-25U and LP-150U, respectively, in the case of *k*_2_ values. These errors were acceptable according to the limit of 20%, which was mentioned in Section 3.6. These models, in combination with the predicted models of the microbial growth rates, are prerequisites for developing chill chain management tools based on TTI in order to monitor the time–temperature history of the packaged fish products during distribution and storage.

## 4. Conclusions

The use of CO_2_ emitters serving as active food packaging systems in combination with modified atmosphere packaging inhibited the dominant spoilage microorganisms and improved the chemical freshness (K-values) in gilthead seabream fillets, leading to shelf-life extensions during isothermal and non-isothermal refrigerated storage. The gas concentration (CO_2_ and O_2_) in the package headspace was significantly affected by the storage time, temperature and packaging conditions. Gilthead seabream fillets packed in active packaging with a low initial CO_2_ concentration (20%) presented a significantly reduced bacterial growth rate (TVC, *Pseudomonas* spp., *Enterobacteriaceae* spp. and H_2_S-producing bacteria) and extended shelf-life compared to the MAP samples. The shelf-life of the MAP-PAD and MAP seabream fillets was estimated at 17, 13, 8 and 3 days and 16, 11, 6 and 3 days at 0 °C, 2.5 °C, 5 °C and 10 °C, respectively. Moreover, this study suggests that enzymatic FreshTag TTI consists of methyl myristate as the substrate, and an enzyme concentration of 25 units can be useful as a quality monitoring tool for gilthead seabream fillets packed under modified atmosphere with CO_2_ emitters, while TTI with a mixture of trilaurin and tripalmitin and 150 units of enzyme concentration can effectively be applied in the case of MAP gilthead seabream fillets. At any point during isothermal and non-isothermal storage, which simulated the chill chain condition from production to consumption, the response of the appropriately selected TTI may provide a reliable estimation of the quality and the remaining shelf-life of the MAP gilthead seabream fillets. 

Coupling the use of reliable kinetic models for the shelf-life estimation of fish products with the models of appropriate TTI responses could allow the optimization and better management of the cold chain. Active and smart packaging can be used useful, cost-effective tools for fish aquaculture companies in order to provide extended and controlled quality to the consumers. Both tools should be further studied and used in other fish products (whole, gutted and fillets); species and modified atmosphere packaging conditions.

## Figures and Tables

**Table 1:** Representative microbial population counts (log CFU g^−1^) for the total viable count (TVC), *Pseudomonas* spp., *Enterobacteriaceae* spp. and H_2_S-producing bacteria in the MAP-PAD and MAP seabream fillets at isothermal storage conditions of 0–10 °C.

		Total Aerobic Viable Count (log CFU g^−1^)		*Pseudomonas* spp. (log CFU g^−1^)		*Enterobacteriaceae* spp. (log CFU g^−1^)		H_2_S-producing Bacteria (log CFU g^−1^)	
Storage Temperature	Storage Time	MAP-PAD	MAP	MAP-PAD	MAP	MAP-PAD	MAP	MAP-PAD	MAP
0 °C	0 days	5.41 ± 0.05	5.41 ± 0.05	5.19 ± 0.20	5.19 ± 0.20	4.05 ± 0.15	4.05 ± 0.15	4.23 ± 0.17	4.23 ± 0.17
	14 days	6.74 ± 0.10	7.07 ± 0.13	5.84 ± 0.15	6.48 ± 0.11	4.45 ± 0.34	5.04 ± 0.33	6.63 ± 0.07	7.18 ± 0.04
	24 days	8.06 ± 0.24	8.14 ± 0.02	6.88 ± 0.10	7.04 ± 0.02	6.40 ± 0.20	7.11 ± 0.31	7.89 ± 0.09	8.15 ± 0.15
2.5 °C	0 days	4.54 ± 0.15	4.54 ± 0.15	4.55 ± 0.25	4.55 ± 0.25	2.88 ± 0.04	2.88 ± 0.04	3.49 ± 0.10	3.49 ± 0.10
	12 days	6.48 ± 0.14	6.98 ± 0.14	5.11 ± 0.10	5.76 ± 0.16	4.30 ± 0.05	4.67 ± 0.09	5.97 ± 0.09	5.99 ± 0.03
	25 days *	8.23 ± 0.03	8.13 ± 0.04	5.61 ± 0.10	6.35 ± 0.15	4.74 ± 0.14	4.89 ± 0.03	7.04 ± 0.02	7.01 ± 0.05
5 °C	0 days	5.37 ± 0.05	5.37 ± 0.05	5.19 ± 0.20	5.19 ± 0.20	4.05 ± 0.15	4.05 ± 0.15	4.23 ± 0.17	4.23 ± 0.17
	7 days	6.77 ± 0.13	6.81 ± 0.09	6.16 ± 0.18	6.74 ± 0.04	4.71 ± 0.27	5.95 ± 0.05	5.82 ± 0.16	6.96 ± 0.16
	14 days	8.38 ± 0.09	8.45 ± 0.15	6.71 ± 0.03	6.77 ± 0.07	7.21 ± 0.03	7.00 ± 0.05	7.68 ± 0.04	7.95 ± 0.15
10 °C	0 days	5.35 ± 0.11	5.35 ± 0.11	5.19 ± 0.20	5.19 ± 0.20	4.05 ± 0.15	4.05 ± 0.15	4.23 ± 0.17	4.23 ± 0.17
	3 days	7.32 ± 0.05	7.43 ± 0.03	6.30 ± 0.02	6.77 ± 0.03	5.50 ± 0.05	7.02 ± 0.05	7.16 ± 0.02	7.37 ± 0.03
	8 days	8.75 ± 0.11	8.91 ± 0.10	6.88 ± 0.02	7.52 ± 0.04	7.54 ± 0.12	7.61 ± 0.05	7.71 ± 0.29	8.22 ± 0.12

* The end of storage for the MAP-PAD samples was on day 33 compared to the MAP samples, were the experiment ended on day 25.

**Table 2:** Kinetic parameters, i.e., growth rate (k, in d^−1^), lag phase (λ, in d) and final microbial population (N_max_), according to the Baranyi growth model, for the total viable count (TVC), *Pseudomonas* spp., *Enterobacteriaceae* spp. and H_2_S-producing bacteria in seabream fillets at isothermal storage conditions at 0–10 °C.

Storage Temperature	0 °C	2.5 °C	5 °C	10 °C
Total aerobic viable count (TVC)				
MAP-PAD				
Growth rate, *k* (d^−1^)	0.224 ± 0.027 ^a^	0.294 ± 0.020 ^ab^	0.51 ± 0.14 ^b^	1.07 ± 0.23 ^c^
Lag phase, *λ* (d)	8.44 ± 0.97 ^x^	2.88 ± 0.37 ^y^	3.65 ± 0.58 ^y^	1.16 ± 0.45 ^y^
N_max_	8.09 ± 0.10	8.48 ± 0.47	8.48 ± 0.23	8.73 ± 0.26
R^2^ fit	0.992	0.982	0.960	0.963
MAP				
Growth rate, *k* (d^−1^)	0.186 ± 0.031 ^a^	0.298 ± 0.052 ^ab^	0.838 ± 0.19 ^b^	1.26 ± 0.187 ^c^
Lag phase, *λ* (d)	6.36 ± 1.52 ^x^	1.52 ± 0.78 ^y^	3.88 ± 0.56 ^y^	1.55 ± 0.25 ^y^
N_max_	8.14 ± 0.29	7.92 ± 1.39	8.61 ± 0.15	8.71 ± 0.14
R^2^ fit	0.953	0.903	0.982	0.989
*Pseudomonas* spp.				
MAP-PAD				
Growth rate, *k* (d^−1^)	0.130 ± 0.016 ^a^	0.257 ± 0.0247 ^b^	0.427 ± 0.037 ^c^	0.629 ± 0.035 ^d^
Lag phase, *λ* (d)	8.76 ± 0.13 ^a^	5.11 ± 0.99 ^a^	4.63 ± 0.18 ^a^	1.09 ± 0.03 ^b^
N_max_	6.83 ± 0.18	5.71 ± 0.06	6.62 ± 0.05	6.86 ± 0.16
R^2^ fit	0.957	0.771	0.985	0.947
MAP				
Growth rate, *k* (d^−1^)	0.143 ± 0.032 ^a^	0.215 ± 0.1232 ^b^	0.416 ± 0.088 ^c^	0.645 ± 0.221 ^d^
Lag phase, *λ* (d)	6.43 ± 1.84 ^a^	5.20 ± 0.34 ^a^	5.94 ± 0.35 ^a^	0.87 ± 0.03 ^b^
N_max_	7.04 ± 0.36	6.35 ± 0.50	6.85 ± 0.17	7.50 ± 0.27
R^2^ fit	0.914	0.843	0.929	0.916
*Enterobacteriaceae* spp.				
MAP-PAD				
Growth rate, *k* (d^−1^)	0.251 ± 0.073 ^a^	0.421 ± 0.007 ^a^	0.543 ± 0.377 ^b^	0.894 ± 0.034 ^c^
Lag phase, *λ* (d)	12.68 ± 2.68 ^a^	7.12 ± 0.80 ^b^	5.82 ± 0.76 ^b^	1.34 ± 0.08
N_max_	6.78 ± 0.19	4.74 ± 0.34	7.23 ± 0.05	7.53 ± 0.04
R^2^ fit	0.866	0.999	0.982	0.999
MAP				
Growth rate, *k* (d^−1^)	0.324 ± 0.072 ^a^	0.433 ± 0.033 ^a^	0.672 ± 0.086 ^b^	1.050 ± 0.090 ^c^
Lag phase, *λ* (d)	11.58 ± 1.68 ^a^	4.76 ± 0.74 ^b^	4.06 ± 0.9 ^b^	-
N_max_	6.95 ± 0.21	4.75 ± 0.04	6.81 ± 0.11	7.60 ± 0.87
R^2^ fit	0.933	0.980	0.984	0.812
H_2_S-producing bacteria				
MAP-PAD				
Growth rate, *k* (d^−1^)	0.183 ± 0.041 ^a^	0.219 ± 0.029 ^a^	0.339 ± 0.130 ^a^	1.050 ± 0.130 ^b^
Lag phase, *λ* (d)	1.33 ± 0.04	-	-	-
N_max_	7.95 ± 1.42	6.94 ± 0.32	7.53 ± 0.55	7.78 ± 0.22
R^2^ fit	0.888	0.967	0.798	0.969
MAP				
Growth rate, *k* (d^−1^)	0.209 ± 0.032 ^a^	0.295 ± 0.043 ^a^	0.695 ± 0.169 ^a^	1.150 ± 0.160 ^b^
Lag phase, *λ* (d)	-	-	-	-
N_max_	8.42 ± 0.76	6.69 ± 0.50	7.43 ± 0.41	8.13 ± 0.23
R^2^ fit	0.922	0.931	0.908	0.964

^a^, ^b^, ^c^ and ^d^ Different superscripts in the same row for the estimated growth rates (k) of the same microorganisms indicate statistically significant differences between different storage temperatures 0–10 °C. ^x^ and ^y^ Different superscripts in the same rows for the lag phase (λ) of the same microorganisms indicate statistically significant differences between different storage temperatures 0–10 °C.

**Table 3:** Activation energy (*E_a_*) estimated by the Arrhenius equation for the bacterial growth rate for the total viable count (TVC), *Pseudomonas* spp. and *Enterobacteriaceae* spp. in gilthead seabream fillets stored under isothermal storage conditions in the range 0–10 °C.

	TVC		*Pseudomonas* spp.		*Enterobacteriaceae* spp.		H_2_S-Producing Bacteria	
	*E_a_* (kJ mol^−1^)	R^2^	*E_a_* (kJ mol^−1^)	R^2^	*E_a_* (kJ mol^−1^)	R^2^	E_a_ (kJ mol^−1^)	R^2^
MAP-PAD	113.1	0.887	93.7	0.918	81.4	0.969	116.2	0.961
MAP	133.9	0.883	100.5	0.952	90.3	0.982	120.4	0.943

**Table 4:** Shelf-life (*t_SL_*) in days of MAP-PAD and MAP seabream fillets stored in isothermal conditions (0–10 °C). The acceptability limit for the shelf-life estimation was 7 log CFU g^−1^ for the total viable count.

Temperature	Shelf-Life *t_SL_* (Days)	
	MAP-PAD	MAP
0 °C	17	16
2.5 °C	13	11
5 °C	8	6
10 °C	3	3

**Table 5:** Response rate constants *k*_1_ and *k*_2_ for all the examined M-type TTIs at 0–15 °C.

ΤΤΙ	Temperature (°C)	0	2.5	5	10	15
M-5U	*k*_1_ (d^−1^)	50.4	37.2	29.2	10.2	6.90
	s. error (k_1_)	1.91	1.24	0.43	0.32	0.02
	*k*_2_ (d^−1^)	7.83	4.07	3.20	1.48	1.24
	s. error (k_2_)	1.60	0.94	0.31	0.26	0.02
M-10U	*k*_1_ (d^−1^)	31.3	23.1	14.0	6.85	3.97
	s. error (k_1_)	0.36	0.10	0.37	0.02	0.04
	*k*_2_ (d^−1^)	6.18	2.37	3.65	0.57	0.59
	s. error (k_2_)	0.44	0.09	0.35	0.02	0.04
M-15U	*k*_1_ (d^−1^)	22.3	18.1	12.3	5.57	3.11
	s. error (k_1_)	0.36	0.18	0.25	0.10	0.02
	*k*_2_ (d^−1^)	4.79	2.76	2.25	1.06	0.48
	s. error (k_2_)	0.26	0.16	0.24	0.08	0.01
M-20U	*k*_1_ (d^−1^)	19.2	14.9	11.9	3.69	2.47
	s. error (k_1_)	0.36	0.18	0.25	0.10	0.02
	*k*_2_ (d^−1^)	4.95	2.02	1.68	0.64	0.50
	s. error (k_2_)	0.26	0.16	0.24	0.08	0.01
M-25U	*k*_1_ (d^−1^)	15.5	12.5	8.03	2.96	1.63
	s. error (k_1_)	0.34	0.49	0.13	0.03	0.02
	*k*_2_ (d^−1^)	2.99	2.57	1.49	0.60	0.21
	s. error (k_2_)	0.28	0.33	0.12	0.03	0.02
M-50U	*k*_1_ (d^−1^)	8.11	5.47	3.77	1.79	0.93
	s. error (k_1_)	0.07	0.17	0.03	0.03	0.01
	*k*_2_ (d^−1^)	1.39	1.24	0.47	0.34	0.16
	s. error (k_2_)	0.08	0.10	0.02	0.02	0.01
M-75U	*k*_1_ (d^−1^)	6.03	3.99	2.73	0.97	0.68
	s. error (k_1_)	0.03	0.04	0.02	0.02	0.01
	*k*_2_ (d^−1^)	0.86	0.65	0.27	0.17	0.15
	s. error (k_2_)	0.03	0.03	0.02	0.01	0.01
M-100U	*k*_1_ (d^−1^)	4.26	3.12	2.06	0.97	0.46
	s. error (k_1_)	0.03	0.07	0.02	0.02	0.01
	*k*_2_ (d^−1^)	0.58	0.53	0.31	0.09	0.08
	s. error (k_2_)	0.03	0.07	0.01	0.01	0.01

**Table 6:** Response rate constants *k*_1_ and *k*_2_ for all the examined LP-type TTIs at 0–15 °C.

ΤΤΙ	Temperature (°C)	0	2.5	5	10	15
LP-100U	*k*_1_ (d^−1^)	33.1	14.0	7.90	3.15	1.15
	s. error (k_1_)	0.33	0.42	0.13	0.06	0.02
	*k*_2_ (d^−1^)	10.9	4.26	1.84	0.79	0.27
	s. error (k_2_)	0.25	0.31	0.12	0.06	0.02
LP-150U	*k*_1_ (d^−1^)	21.4	12.1	8.77	1.81	0.77
	s. error (k_1_)	0.80	0.16	0.15	0.04	0.03
	*k*_2_ (d^−1^)	8.60	2.09	2.52	0.57	0.18
	s. error (k_2_)	0.79	0.14	0.13	0.03	0.03
LP-300U	*k*_1_ (d^−1^)	11.3	3.49	1.38	0.85	0.30
	s. error (k_1_)	0.40	0.14	0.04	0.02	0.03
	*k*_2_ (d^−1^)	3.49	0.86	0.38	0.23	0.06
	s. error (k_2_)	0.36	0.14	0.04	0.01	0.03
LP-500U	*k*_1_ (d^−1^)	9.87	4.03	1.75	0.49	0.20
	s. error (k_1_)	0.10	0.06	0.03	0.01	0.01
	*k*_2_ (d^−1^)	2.25	1.01	0.41	0.14	0.05
	s. error (k_2_)	0.09	0.06	0.02	0.01	0.01

**Table 7:** Estimation of the parameters of the total mathematical models (Equation (4)) for M-type and LP-type TTIs.

Parameter	M-Type	LP-Type
*E_a_* (kJ/mol)	97.1	148.0
*k_1ref(c=1U)_* (d^−1^)	128.6	1822.4
*k_2ref(c=1U)_* (d^−1^)	25.7	298.1
A	0.861	1.10
Β	0.861	0.970
R^2^	0.970	0.967

**Table 8:** Kinetic parameters (microbial growth rate (*k*), lag phase (*λ*) and maximum bacterial population counts (N_max_)) of the Arrhenius model for the bacterial growth rate for the total viable count (TVC), *Pseudomonas* spp., *Enterobacteriaceae* spp. and H_2_S-producing bacteria in seabream fillets at non-isothermal conditions (T_eff_ = 4.8 °C).

	MAP-PAD	MAP
*TVC*		
Growth rate, *k* (d^−1^)	0.348 ± 0.015	0.551 ± 0.073
Lag phase, *λ* (in d)	3.76 ± 0.96	3.18 ± 0.57
N_max_	8.52 ± 0.14	8.45 ± 0.44
R^2^ fit	0.918	0.987
*Pseudomonas* spp.		
Growth rate, *k* (d^−1^)	0.355 ± 0.041	0.367 ± 0.049
Lag phase, *λ* (in d)	-	-
N_max_	6.18 ± 0.30	6.36 ± 0.16
R^2^ fit	0.801	0.931
*Enterobacteriaceae* spp.		
Growth rate, *k* (d^−1^)	0.493 ± 0.0282	0.510 ± 0.025
Lag phase, *λ* (in d)	2.85 ± 0.05	3.85 ± 0.45
N_max_	5.35 ± 0.47	6.11 ± 0.25
R^2^ fit	0.798	0.951
H_2_S-producing bacteria		
Growth rate, *k* (d^−1^)	0.276 ± 0.043	0.467 ± 0.047
Lag phase, λ (in d)	-	-
N_max_	7.12 ± 0.23	7.81 ± 0.20
R^2^ fit	0.950	0.978

**Table 9:** Predicted growth rates (d^−1^) and relative errors (RE%) of the total viable count (TVC), *Pseudomonas* spp., *Enterobacteriaceae* spp. and H_2_S-producing bacteria for gilthead seabream fillets stored under non isothermal conditions, calculated by the developed Arrhenius-type models.

*k_predicted_* (d^−1^)	Total Viable Count	*Pseudomonas* spp.	*Enterobacteriaceae* spp.	H_2_S-Producing Bacteria
MAP-PAD	0.398 ± 0.024 RE% = 14%	0.244 ± 0.012 RE% = 31%	0.401 ± 0.015 RE% = 18%	0.351 ± 0.025 RE% = 27%
MAP	0.524 ± 0.046 RE% = 5%	0.321 ± 0.009 RE% = 12%	0.486 ± 0.023 RE% = 5%	0.447 ± 0.0017 RE% = 2%

## Data Availability

The data are available from the corresponding author.
